# Estimating preclinical amyloid positivity in the community: a case study transporting estimates from ADNI to ARIC

**DOI:** 10.1002/alz.089765

**Published:** 2025-01-09

**Authors:** Emma K Stapp, Elizabeth Rose Mayeda, Elizabeth A Stuart, Rebecca F. Gottesman, Dean F. Wong, Thomas H. Mosley, Michael E. Griswold, M. Maria Glymour, Scott C Zimmerman, Melinda C Power

**Affiliations:** ^1^ The George Washington University, Washington, DC USA; ^2^ University of California, Los Angeles Fielding School of Public Health, Los Angeles, CA USA; ^3^ Johns Hopkins University, Baltimore, MD USA; ^4^ National Institute of Neurological Disorders & Stroke Intramural Research Program, National Institute of Health, Bethesda, MD USA; ^5^ Washington University in St. Louis School of Medicine, St. Louis, MO USA; ^6^ University of Mississippi Medical Center, Jackson, MS USA; ^7^ University of Mississippi Medical Center, The MIND Center, Jackson, MS USA; ^8^ Boston University School of Public Health, Boston, MA USA; ^9^ University of California, San Francisco, San Francisco, CA USA

## Abstract

**Background:**

Estimates of the prevalence of preclinical amyloid positivity in the US general population are of great interest to the field, but difficult to measure and thus unavailable in representative studies. A statistical approach from causal inference, ‘transport’, may allow for improved generalizability of findings from a sample of persons from one population to another. We aimed to explore the feasibility and validity of extending results from a deeply‐phenotyped convenience sample, the Alzheimer’s Disease Neuroimaging Initiative (ADNI), to a representative target sample, the Atherosclerosis Risk in Communities Study PET Amyloid Imaging Study (ARIC‐PET) — with “proof of concept” defined by the performance of the transport estimator in recovering the observed prevalence of amyloid positivity in ARIC‐PET.

**Method:**

Eligible ARIC‐PET and ADNI participants were either white or Black and had normal cognition or mild cognitive impairment. Amyloid positivity was defined using study‐specific cutoffs for standardized uptake value ratio (SUVR). Probability of selection into ADNI (vs. into ARIC‐PET), given harmonized sociodemographic and clinical covariates, was estimated using gradient boosted trees (GBM). The resulting study participation probability scores were used to transport the prevalence of amyloid positivity from ADNI to ARIC using inverse odds of sampling weights. Estimates of amyloid positivity prevalence derived from ADNI and from transporting from ADNI to ARIC‐PET were compared to the observed prevalence in ARIC‐PET, overall and by age, sex, race, education, and APOE e4 status.

**Result:**

Transported prevalences substantially underestimated observed prevalences of amyloid positivity in most groups, with the exception of white, non‐Hispanic individuals and persons with at least one APOE e4 allele, where prevalence was slightly overestimated. Prevalence of preclinical amyloid positivity based on transporting ADNI was further from the prevalence in ARIC‐PET than the raw prevalence of amyloid positivity in ADNI except for white, non‐Hispanic individuals and those with mild cognitive impairment.

**Conclusion:**

Transport estimators rely on adequately modeling the selection process. Available data may be insufficient to capture selection biases into deeply‐phenotyped convenience samples, particularly for groups who are under‐represented in research. Transport estimators will have greatest utility when applied to samples derived from known sampling strategies, and with increased diversity in dementia research.

